# Nuclear Interactions: A Spotlight on Nuclear Mitochondrial Membrane Contact Sites

**DOI:** 10.1177/25152564221096217

**Published:** 2022-05-03

**Authors:** Jana Ovciarikova, Shikha Shikha, Lilach Sheiner

**Affiliations:** 1 Wellcome Centre for Integrative Parasitology, 3526 University of Glasgow, UK

**Keywords:** membrane contact sites, mitochondrion (mitochondria), nucleus, parasite

## Abstract

Membrane contact sites (MCS) are critical for cellular functions of eukaryotes, as they enable communication and exchange between organelles. Research over the last decade unravelled the function and composition of MCS between a variety of organelles including mitochondria, ER, plasma membrane, lysosomes, lipid droplets, peroxisome and endosome, to name a few. In fact, MCS are found between any pair of organelles studied to date, with common functions including lipid exchange, calcium signalling and organelle positioning in the cell. Work in the past year has started addressing the composition and function of nuclear-mitochondrial MCS. Tether components mediating these contacts in yeast have been identified via comprehensive phenotypic screens, which also revealed a possible link between this contact and phosphatidylcholine metabolism. In human cells, and in the protozoan parasites causing malaria, proximity between these organelles is proposed to promote cell survival via a mitochondrial retrograde response. These pioneering studies should inspire the field to explore what cellular processes depend on the exchange between the nucleus and the mitochondrion, given that they play such central roles in cell biology.

Cellular compartmentalisation into organelles is an essential trait of eukaryotes which enables multi-level control of critical cellular functions. This role has two faces: on one hand organelles act as a separate biochemical microenvironment that host distinct pathways. On the other hand, communication and exchange between organelles is required to enable shared biosynthetic pathways, inter-organelle signalling and organelle positioning in the cell. These essential interactions between organelles are mediated by the so-called Membrane Contact Sites (MCS). Over the past decade the cell biology field had seen a growing focus on the composition and function of these sites. It seems that MCS function between any two organelles studied to date, and in some cases the same organelle pair has different types of MCS mediated by different tethers and performing different functions. Over the past year a new contact has sprung to the attention of the cell biology field: that of the nucleus and the mitochondrion (nmMCS) which we review herein.

The first MCS tether identified (Kornmann et al., 2009), and likely the MCS mediated interaction best characterised at the molecular level, is the one between the ER and mitochondrion. ER-mitochondrial MCS functions include calcium exchange, lipid exchange and control of mitochondrial dynamics, of autophagy and of apoptosis. These functions and the corresponding contacts are mediated by numerous identified tethers (reviewed here [Lin et al., 2021]). The nuclear envelope (NE) is contiguous with the ER, with some similarities such as the presence of ribosomes embedded in the cytosol facing surface. However, there are numerous proteins enriched in the NE compared to the peripheral and even perinuclear ER (Cheng et al., 2019; Tang et al., 2020), that render the NE and ER membranes distinct sub-domains by composition, in addition to their spatial distinction. Moreover, the membrane fraction that contains physically associated ER and mitochondria – mitochondrial associated membranes (MAMs) – whose molecular, biochemical, and metabolic nature has been characterised in detail (Csordás et al., 2006; Rusiñol et al., 1994), are mostly formed by peripheral ER tubules. Thus, it is expected that the nmMCS would be spatially, biochemically, and functionally separate from the ER-mitochondria MCS studied to date, which merits their study and consideration of their function as a separate cellular feature.

As is the case for many types of MCS, the proximity between mitochondrion and nucleus shown by imaging has been well documented for decades prior to its investigation in the context of MCS composition and function (Miller et al., 1995; Prachar, 2003). Yet, while membrane proximity is a pre-requisite to MCS formation, it does not in itself imply the presence of a functional contact. MCS are defined by four consensus features (Scorrano et al., 2019): first, despite the close proximity between the membranes, there is no fusion; second, the membranes are actively held together by tether molecules; third, there is always a function that necessitates the contact; and, lastly, partially as a result of the previous points, there is a specific characteristic proteome and/or lipidome for the MCS. With these criteria in mind, a recent study set out to explore the molecular detail of nmMCS in the yeast *Saccharomyces cerevisiae,* which led to the identification of the first nmMCS tether (Eisenberg-Bord et al., 2021). In this study, the authors first generated a “nmMCS marker” using their well-established split fluorescence MCS reporter system (Shai et al., 2018). Observations from two screens were then combined to identify tether component candidates: in one screen the authors integrated the nmMCS marker into an mCherry library. This allowed the identification of proteins that co-localize with the nmMCS reporter signal (57 hits). The other screen sought genes whose over-expression enhanced the nmMCS signal obtained with the new reporter which narrowed down a list of 12 hits (Figure 1). One of the candidates that emerged from both screens is a nuclear protein which was named Cnm1, for Contact Nucleus Mitochondria 1. In line with being a tether component, over-expression of Cnm1 resulted in mitochondrial crowding around the nucleus, indicating that this protein can mediate active recruitment of mitochondria to be in contact with the nucleus; this observation was called as “clustering” by the authors. This phenotype provided the authors with a tool to identify additional factors involved in the Cnm1 contact sites: they screened for mutants that reverse or reduce the contact mediated mitochondria clustering induced by the over-expression of Cnm1. The only candidate resulting from this “clustering-loss” screen, known to be a mitochondrial protein is a component of the mitochondrial outer-membrane protein import translocon, Tom70. A series of experiments provided support for the interaction of Cnm1 and Tom70 at the contact: Immunoprecipitation experiment confirmed a general interaction between those two proteins; deletion of Tom70 affected the nuclear distribution of Cnm1; overexpression of Cnm1 resulted in localisation of an artificial, soluble, Tom70 to the nucleus; and likewise, an artificial and soluble Cnm1 accumulates around the mitochondria upon overexpression of Tom70. Taken together these observations suggest that Tom70 and Cnm1 may act together as an nmMCS tether in yeast (Figure 2). Whether other nmMCS tethers exist in yeast remains to be discovered and some interesting candidate emerging from the screens might provide leads for future studies of this intriguing question.

**Figure 1.**
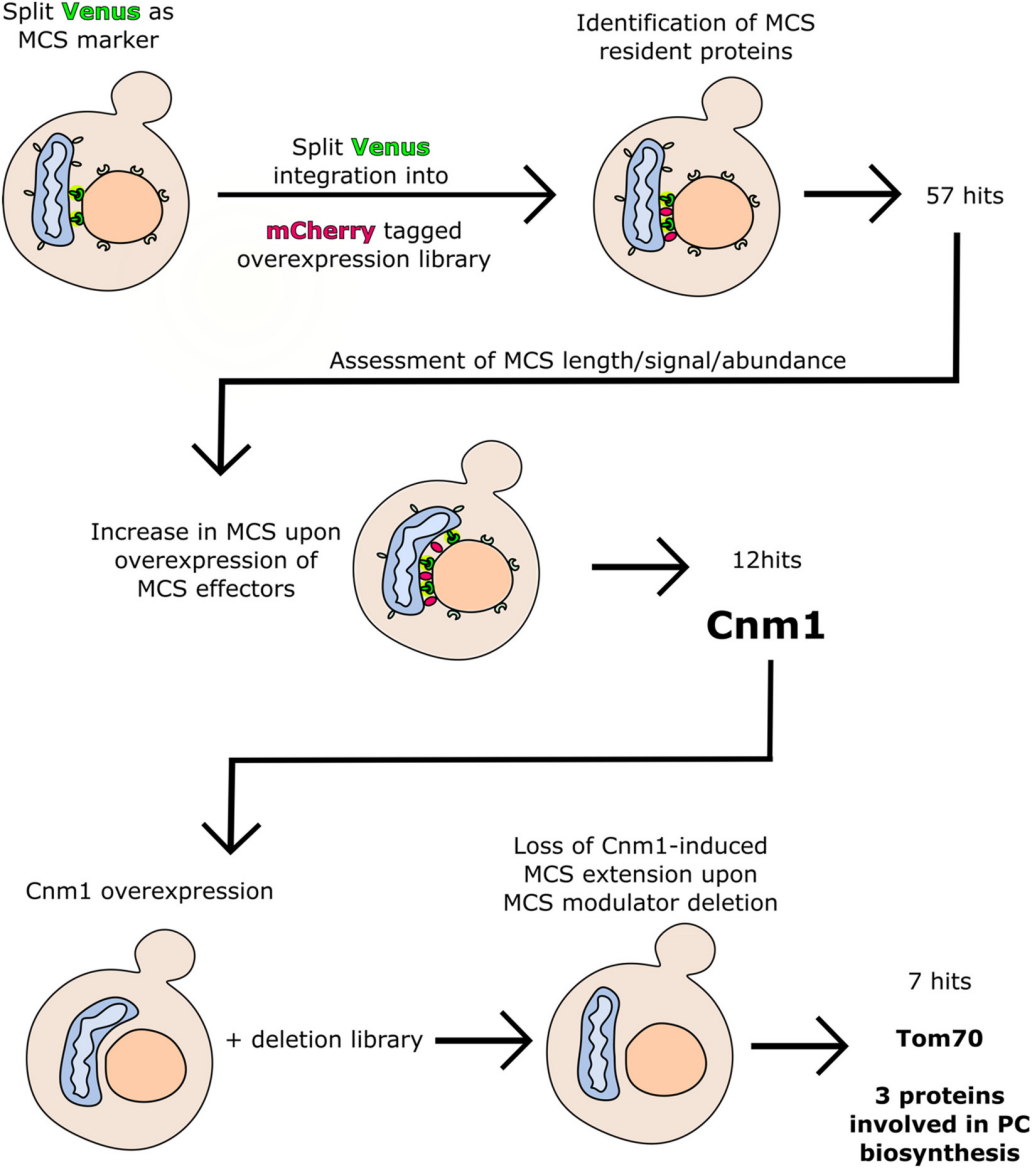
A summary of the pipeline of screens leading to the identification of Cnm1 mediated nmMCS in yeast. The schemes depict the split Venus based nmMCS marker in green, mCherry tagged proteins in red, nucleus in orange and mitochondrion in blue. The mitochondrion is seen in two shapes: one that represent wild type and is positioned near the nucleus, and one that represent the contact-mediated mitochondrial clustering around the nucleus phenotype and is bent around the nucleus. The number of hits from each step of the study is indicated, and key hits that were followed up are mentioned in bold.

**Figure 2.**
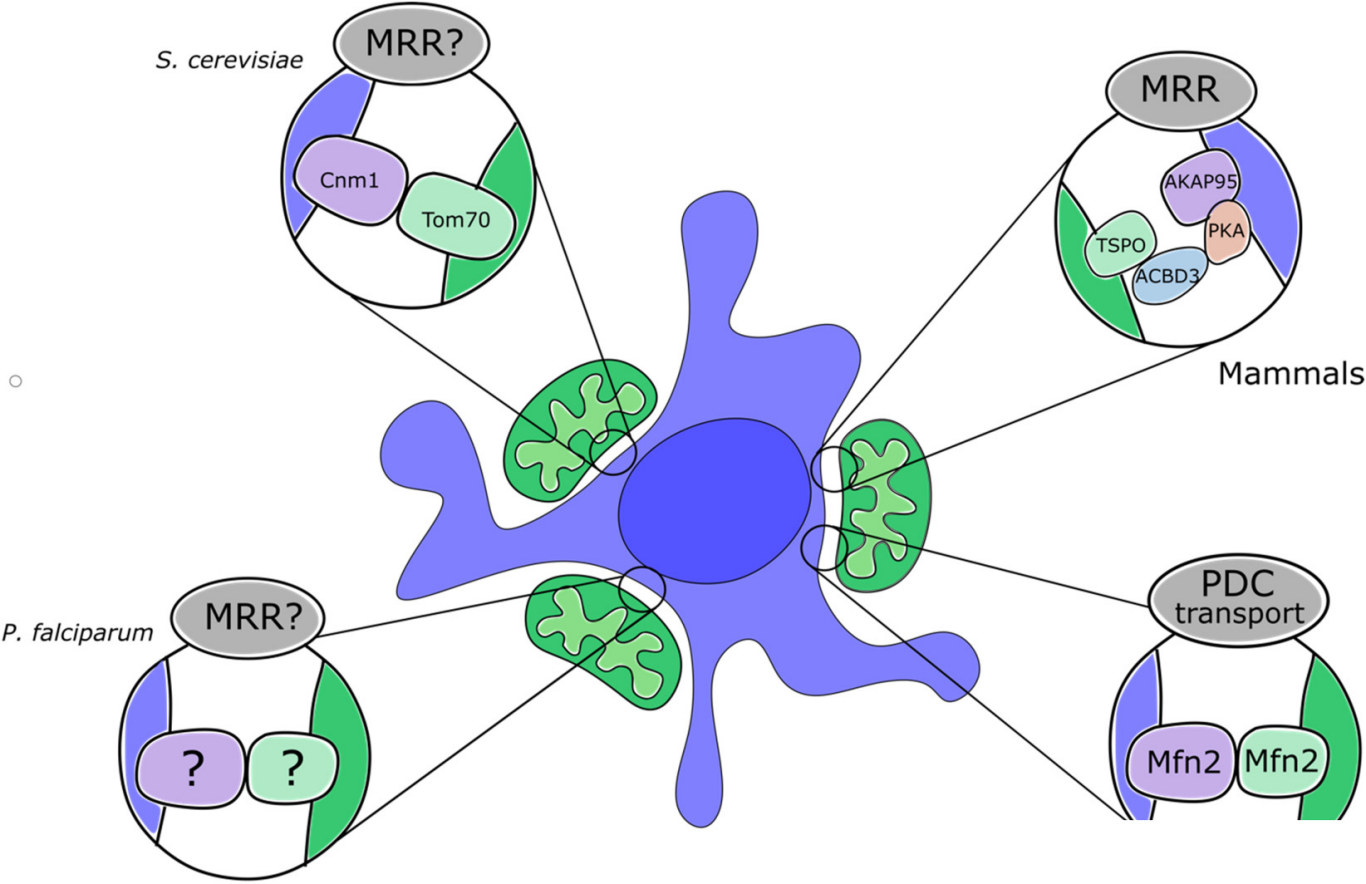
A schematic representation of recently reported nmMCS highlighting the proposed tether components and functions.

An additional observation that emerged from the screen of mutants leading to reduced contact-mediated mitochondrial-nuclear clustering, is the independent identification of three different components of the phosphatidylcholine (PC) biosynthesis pathway. This finding points to a possible link between the Cnm1 mediated nmMCS and PC metabolism. In support of such a link, disruption of the PC biosynthesis pathway affects Cnm1 levels as well as the extent of the observed nmMCS. Whether this new nmMCS directly regulates PC metabolism and through what mechanism remains to be studied.

The new focus on nmMCS raises the exciting question of what other cellular functions may be supported or controlled through an exchange between those two organelles. Two recent papers describe enhanced proximity between mitochondria and nucleus that is linked to stress resistance and cell survival. Mitochondria are known to play an active role in reprogramming of cells, whereby mitochondrial damage is communicated to the nucleus leading to change in gene transcription. Since the traditional way of thinking about eukaryotic cell biology is that signals move from the nucleus to the rest of the cell, this mitochondrial to nuclear communication is often referred to as mitochondrial retrograde response (MRR). Both studies postulate a putative role for nmMCS in supporting MRR (Connelly et al., 2021; Desai et al., 2020).

The first study hypothesised that nmMCS might catalyse MRR in the context of pro-survival pathways in cancer cells, via the mediation of cholesterol, reactive oxygen species and calcium (Connelly et al., 2021). The authors focused on the outer membrane translocator protein (TSPO) as a key candidate for tethering. TSPO was selected due to its role in repressing mitophagy, its binding of cholesterol, and an observed TSPO overexpression in cells that are resistant to chemotherapy. The authors showed enhanced proximity between mitochondria and nucleus in a cancer cell line resistant to treatment and highlighted the presence of TSPO in these proximity areas. They further provided additional evidence for a correlation between TSPO overexpression or downregulation and mitochondria coalescing or releasing from the nucleus respectively. Adding to that, TEM analysis of the MRR-induced cells provided further evidence to the proximity of nucleus and mitochondria, with the distance between the two organelles going below 30nm; this observation was further verified by detection of an accumulated nuclear envelope protein in the mitochondrial fractions under the same conditions. A search for potential tethering partners raised interactors of TSPO with membrane anchoring capacity, leading to the hypothesis that a multiprotein complex is formed between TSPO and its interactors, the A-kinase anchoring protein acyl–coenzyme A binding domain containing 3 (ACBD3) and the protein kinase A (PKA), and the A-kinase- anchoring protein AKAP95, which tethers mitochondria to the nucleus. In support of this hypothesis, co-immunoprecipitation experiments provided evidence for an interaction between TSPO and AKAP95, which was abolished upon depletion of ACBD3 resulting in reduced mitochondrial-nuclear association. These findings represent a putative tether (Figure 2), however further studies would be needed to strengthen this proposal of a putative tether, and to examine in an unbiased way if other components might be involved.

The second study focuses on the eukaryotic unicellular parasite *Plasmodium falciparum*, the causative agent of malaria (Connelly et al., 2021). This study was aimed to identify cellular changes in malaria parasites that persist after treatment with the widely used anti-malarial dihydroartemisinin (DHA). The study showed that these persister cells have enlarged mitochondria with enhanced proximity to the nucleus, detected via fluorescence signals. Due to previous work suggesting mitochondria as a sensor of the cellular damage produced by DHA following reactive oxygen species induced damage, the authors hypothesise that the observed mitochondrial morphological changes and nuclear proximity are linked to this mechanism. Further, in light of the above summarised findings reported in cancer cells, the authors hypothesise that the mitochondrial nuclear proximity might promote a survival response in *Plasmodium* too. Importantly, the proximity seen in this study describes a distance between the contacting membranes that is larger than most MCS described to date, and the observed proximity is yet to be analysed by electron microscopy. Furthermore, no tether has been proposed to mediate this contact. Thus, while the proposed function for a putative nmMCS in *Plasmodium* is intriguing, the existence of a bona fide nmMCS in this organism remains to be fully validated.

Finally, a recent study aiming to understand routes of translocation of the mitochondrial pyruvate dehydrogenase complex into the nucleus, pointed to another potential nmMCS (Zervopoulos et al., 2022). In this study, focused on human cells, the authors first showed that proliferative stimuli such as exposure to serum and to epidermal growth factor (EGF), lead to the crowding of mitochondria around the nucleus. The authors further showed that signal from the mitochondrial protein mitofusin-2 (MFN2) co-localized not only with mitochondria and ER, as previously reported, but also with the nuclear envelope, and that this NE-overlapping signal is enhanced under proliferative stimuli (Zervopoulos et al., 2022). This led to the hypothesis that MFN2 mediated the observed mitochondrial gathering at the nucleus in respond to these stimuli. In support of this hypothesis, isolated mitochondria from wild type cells, were able to tether nuclei isolated from MFN2 depleted cell *in vitro* (Zervopoulos et al., 2022). These observations point to an MFN2 mediated nmMCS that plays a role in the process of cellular response to proliferative stimuli. Interestingly, when put together, these three studies paint a picture whereby nmMCS are involved in facilitating cell proliferation and survival. It will be of interest to see if this represents a universal trend in cell biology.

In conclusion, the new focus on nmMCS should inspire the field to explore what cellular functions may be served through the exchange between those organelles. The critical role of mitochondria in controlling cell fate, triggered the three studies summarised above to hypothesise a role in mediating pro-survival and proliferative signalling. Moreover, the authors of the study performed in cancer cells also raise the important point that mitochondria-produced reactive oxygen species, whose rate of diffusion in the cytosol is slow, would gain a “fast-track” for nuclear accumulation via the nmMCS. This rationale provides further support for a role in retrograde signalling and is in line with how other MCS work. One example for MCS mediated proximity that enhances the natural mobility rate of a signal is the case of calcium exchanged between the ER and the mitochondrion at the porin mediated contact (Modesti et al., 2021). This contact which is formed through interaction between the mitochondrial porin Voltage Dependent Anion Channel (VDAC) and the ER resident inositol trisphosphate receptor (IP3R), creates local high calcium concentration, thus facilitating calcium mobility into mitochondria via the mitochondrial calcium uniporter MCU that has low affinity to calcium (Modesti et al., 2021). Another example for this MCS mode of action is represented by redox nanodomains that are induced at ER-mitochondrial contacts (Booth et al., 2016). Mitochondrial respiration generates H_2_O_2_ which, if it accumulates, is damaging to the cell, and its elimination by degradation and diffusion to the mitochondrial matrix or to the cytoplasm is slower than the rates of its production. It was shown that the calcium uptake at ER-mitochondria MCS mediates H_2_O_2_ release via aligned cristae junction at the contact. The suggested mechanism is that H_2_O_2_ generated by respiration in the cristae space, along with calcium uptake, induces a compression of the cristae which forces their volume through the aligned cristae junctions and ER-mitochondrial contact to the interface between the two organelles (Booth et al., 2016). Thus, the proposed role for nmMCS in facilitating signal mobility is well in line with roles described previously for other MCSs. But what other functions might be served by the interaction of the nucleus and mitochondria? An interesting possibility not yet explored is an exchange in the opposite direction: could nmMCS provide a direct route for the nucleus to govern mitochondrial functions?

The identification of tethering components is a critical step in defining MCS, as it provides means to study function, via genetic manipulation and phenotypic analysis. The identity of tethers, or of other proteins that affect the abundance of the MCS, could also provide hints about function, as demonstrated by the Cnm1 study, where involvement of the nmMCS in PC metabolism is exposed as a possibility. Interestingly, Cnm1 is a yeast specific protein, highlighting a likely case of organism specific MCS. This finding thus adds an example to a growing body of evidence supporting a dogma whereby MCS are highly divergent between different organisms and is in agreement with the hypothesis that MCS evolved independently in different lineages (Wideman & Munoz-Gomez, 2016). The ER-mitochondrion tether ERMES was one of the first examples of organism specific contact (Kornmann et al., 2009). A more recent example for this divergence in MCS is provided by studies of the ER-mitochondrion tether mediated through the mitochondrial porin, VDAC (Figure 3). As mentioned above, in mammalian cells, VDAC partners with the ER-localised IP3R via the chaperone grp75 to mediate a MCS that controls calcium mobilisation from the ER into the mitochondrion (Szabadkai et al., 2006). A study of the divergent protozoan parasites *Toxoplasma gondii,* provided evidence that points at a VDAC mediated ER-mitochondrial MCS, however in this organism VDAC depletion has no effect on calcium homeostasis. Moreover, IP3R is not found in *Toxoplasma* suggesting a different ER partner for this MCS (Mallo et al., 2021). Interestingly, in *Trypanosoma brucei*, another divergent protozoan found in a different eukaryotic clade to that of mammals and yeast, and to the clade of *Toxoplasma*, VDAC and IP3R function as tether between the mitochondrion and the acidocalcisome rather than the ER (Chiurillo et al., 2020; Docampo & Huang, 2021). Thus, the observations from the three unrelated systems suggest that VDAC mediated mitochondria contacts may assume different roles and different composition in divergent organisms, in support of independent evolution of these contacts in each lineage despite VDAC being universal. TSPO, the proposed tether in the cancer study, is also universally conserved. It would be interesting to find out if it plays a role in nmMCS in other organisms and if so, what the partners are.

**Figure 3.**
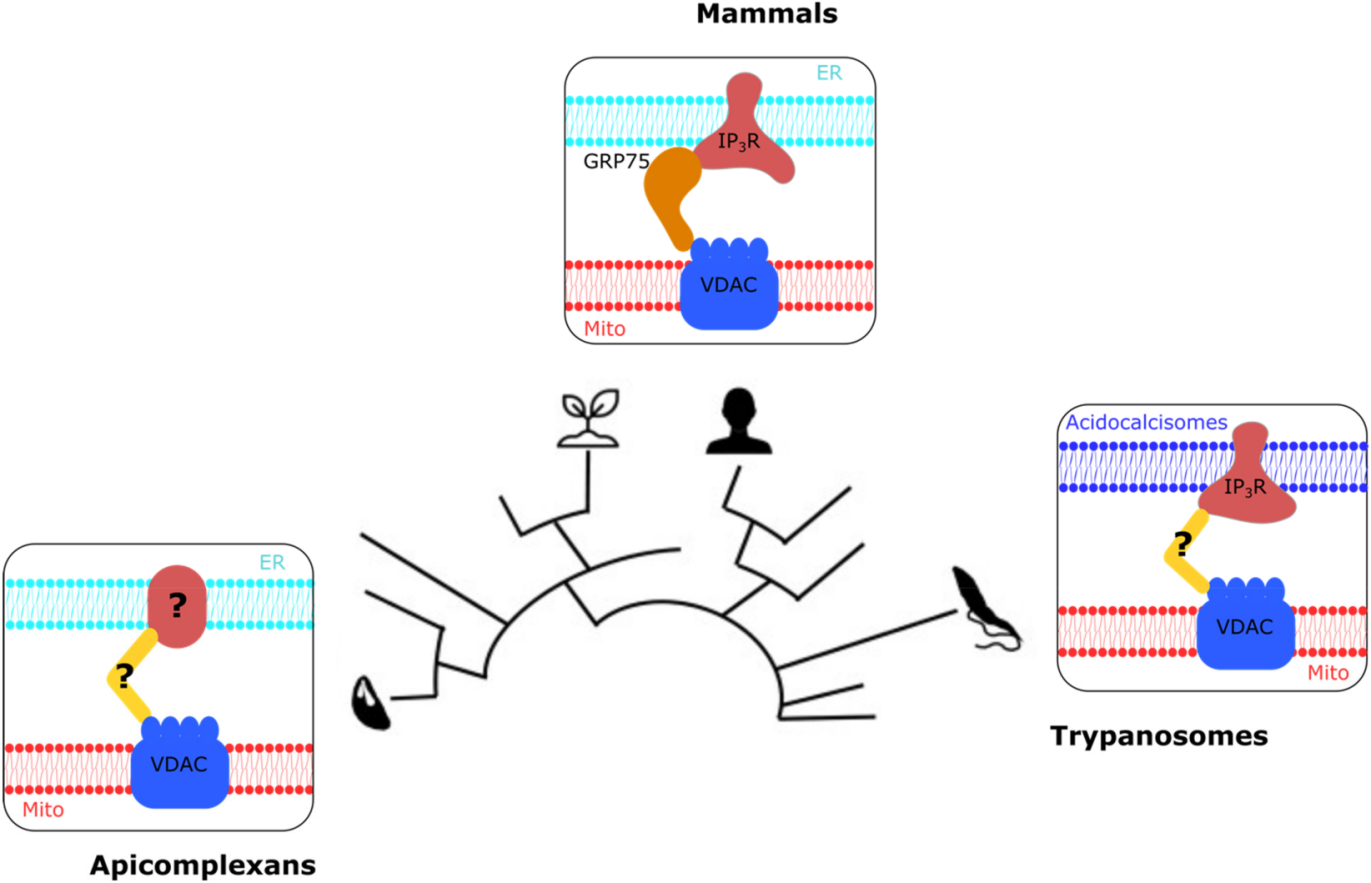
A scheme summarising the different MCS mediated by VDAC in divergent organisms. The three VDAC contacts studied depicted near the branch of the eukaryotic tree to which the corresponding organism belongs. The membranes of the organelles involved are depicted as lipid-bilayer icons, with the name of organelle mentioned. The tethers are depicted as blue (VDAC), yellow (soluble mediator) and red (ER or acidocalcisome tethering partner) shapes with name of the protein mentioned where known.
